# Differentiating Radiation-Induced Necrosis from Recurrent Brain Tumor Using MR Perfusion and Spectroscopy: A Meta-Analysis

**DOI:** 10.1371/journal.pone.0141438

**Published:** 2016-01-07

**Authors:** Ming-Tsung Chuang, Yi-Sheng Liu, Yi-Shan Tsai, Ying-Chen Chen, Chien-Kuo Wang

**Affiliations:** Department of Diagnostic Radiology, National Cheng Kung University Hospital, Tainan, Taiwan; University Medical Center (UMC) Utrecht, NETHERLANDS

## Abstract

**Purpose:**

This meta-analysis examined roles of several metabolites in differentiating recurrent tumor from necrosis in patients with brain tumors using MR perfusion and spectroscopy.

**Methods:**

Medline, Cochrane, EMBASE, and Google Scholar were searched for studies using perfusion MRI and/or MR spectroscopy published up to March 4, 2015 which differentiated between recurrent tumor vs. necrosis in patients with primary brain tumors or brain metastasis. Only two-armed, prospective or retrospective studies were included. A meta-analysis was performed on the difference in relative cerebral blood volume (rCBV), ratios of choline/creatine (Cho/Cr) and/or choline/N-acetyl aspartate (Cho/NAA) between participants undergoing MRI evaluation. A χ^2^-based test of homogeneity was performed using Cochran’s Q statistic and I^2^.

**Results:**

Of 397 patients in 13 studies who were analyzed, the majority had tumor recurrence. As there was evidence of heterogeneity among 10 of the studies which used rCBV for evaluation (Q statistic = 31.634, I^2^ = 97.11%, P < 0.0001) a random-effects analysis was applied. The pooled difference in means (2.18, 95%CI = 0.85 to 3.50) indicated that the average rCBV in a contrast-enhancing lesion was significantly higher in tumor recurrence compared with radiation injury (P = 0.001). Based on a fixed-effect model of analysis encompassing the six studies which used Cho/Cr ratios for evaluation (Q statistic = 8.388, I^2^ = 40.39%, P = 0.137), the pooled difference in means (0.77, 95%CI = 0.57 to 0.98) of the average Cho/Cr ratio was significantly higher in tumor recurrence than in tumor necrosis (P = 0.001). There was significant difference in ratios of Cho to NAA between recurrent tumor and necrosis (1.02, 95%CI = 0.03 to 2.00, P = 0.044).

**Conclusions:**

MR spectroscopy and MR perfusion using Cho/NAA and Cho/Cr ratios and rCBV may increase the accuracy of differentiating necrosis from recurrent tumor in patients with primary brain tumors or metastases.

## Introduction

Gliomas are the most common type of primary brain tumor in adults. The severity (or grade) of these lesions is based on their degree of aggressiveness [1,2]. Contrast-enhanced magnetic resonance imaging (CE-MRI) represents the current mainstay for evaluating treatment response in GBM based on the premise that enlarging lesions, and those which enhance, reflect increasing tumor burden, treatment failure, and poor prognosis [2–6]. Unfortunately, irradiating such tumors can induce changes on CE-MRI that mimic tumor recurrence, so called post treatment radiation effect (PTRE) [7]. Pseudoprogression has been primarily reported in patients who underwent radiotherapy for GBM [8]. In another study of 51 patients with high-grade glioma, Hoffman et al. found six patients (12%) with increased computerized tomography (CT) enhancement following radiation, which later disappeared [9,10].

With the advent of more aggressive management of brain tumors, involving new neoadjuvant strategies such as gamma-knife and stereotactic radiosurgery, the imaging appearance and clinical manifestations of radiation necrosis and recurrence from a previously treated brain tumor can be confusingly similar. Differentiating radiation necrosis from recurrent/progressive tumor is an important but challenging task, as the treatment options and prognosis for each is different. To differentiate between the two, surgical biopsy with re-operation is often required to be certain of the diagnosis before further management can be planned.

It is for this reason that recent studies have investigated the use of more advanced imaging methods that are able to monitor physiological and metabolic properties of tumor [2]. These functional imaging techniques include CT perfusion, MR perfusion [11–15], diffusion weighted imaging (DWI)[2, 3], MR spectroscopy [12, 14, 16–22] single-photon emission computed tomography (SPECT) [23, 24], and positron emission tomography (PET) [25–27]. However, each modality has its limitations. Conventional MRI does not provide sufficient information to differentiate delayed radiation effects from tumor recurrence, whereas PET, MR spectroscopy, and other modalities can lead to false positive findings of tumor recurrence [13].

Although the gold standard is still brain biopsy, high levels of choline (Cho) are often observed in areas with high cellular membrane turnover, and relatively increased cerebral blood volume (rCBV) reflects tumor neovascularization [11–15]. N-acetyl aspartate (NAA) is another metabolite found in neurons, and creatine (Cr) is rich in regions active with energy metabolism. A scoring system using these parameters may increase the accuracy of differentiating recurrent tumor tissues from necrosis caused by delayed radiation effects.

The aim of our study was to evaluate the diagnostic effectiveness of MR perfusion and MR spectroscopy in differentiating recurrent tumor from necrosis caused by radiation, based on parameters such as rCBV and ratios of choline/creatine (Cho/Cr) and choline/N-acetyl aspartate (Cho/NAA). We hypothesized that imaging from MR perfusion and MR spectroscopy has the potential to differentiate recurrent or progressive tumor growth from treatment-induced necrosis in brain tissue after radiation therapy.

## Subjects and Methods

### Selection criteria

We included only two-armed (recurrent tumor vs. necrosis) prospective or retrospective studies of patients with primary brain tumors or brain metastasis evaluated using MR perfusion or MR spectroscopy, or both. The study design had to involve at least one of the outcome measures, i.e., relative cerebral blood volume (rCBV), ratio of Cho/Cr and/or ratio of Cho/NAA. Only English language publications were included.

Letters, comments, editorials, case reports, proceedings, and personal communications were excluded. In addition, any study design which did not contain at least one of the quantitative primary or secondary outcome measures was also excluded.

### Search strategy

Searched databases included Medline, Cochrane, EMBASE, and Google Scholar which were searched until March 4, 2015. The reference lists of relevant studies were hand-searched. Keywords used for the search included magnetic resonance spectroscopy/MR spectroscopy, magnetic resonance perfusion/MR perfusion, brain tumors, brain metastasis, recurrence, radiation injury/radiation necrosis.

### Study selection and data extraction

We adhered to PRISMA (Preferred Reporting Items for Systematic Reviews and Meta-Analyses) guidelines (S1 PRISMA Checklist). Studies were identified and assessed by two independent reviewers using the search strategy (S1 File) and selection criteria. Where there was uncertainty regarding eligibility, a third reviewer was consulted.

The following data were extracted from studies that met the inclusion criteria: the name of the first author, year of publication, study design, number of participants in each treatment group, participants’ age and gender, patients’ type, primary and secondary outcomes, as well as time of follow-up.

### Quality assessment

We used the Newcastle-Ottawa Scale to assess the included studies. The Newcastle-Ottawa Scale is a valid tool for evaluating nonrandomized studies with regard to three criteria: patient selection, comparability of study groups, and outcome assessment. Quality assessment was also performed by two independent reviewers and a third reviewer was consulted for any uncertainty [28].

### Outcome measures

The outcomes of interest were rCBV, and ratios of Cho/Cr and Cho/NAA.

### Statistical analysis

Outcomes for this meta-analysis were the difference in rCBV and ratios of Cho/Cr and Cho/NAA between tumor recurrence and radiation necrosis. If the median and interquartile ranges (IQR) were reported in a study, assumption that the median of the outcome variables equal to the mean response would be made and width of the interquartile range would be approximately 1.35 times the standard deviation [28]. If data lacked in mean and standard deviation, median, range, and size of the sample would be used to estimate the mean and variance [29]. The difference in means with 95% CI was calculated for each individual study and for those studies pooled. A χ2-based test of homogeneity was performed and the inconsistency index (I^2^) and Q statistics were determined. If the I^2^ statistic was > 50%, a random-effects model was used. Otherwise, fixed-effects models were employed. Pooled effects were calculated and a two-sided P value < 0.05 was considered statistically significant.

Sensitivity analysis was carried out for the outcomes using the leave one-out approach. Publication bias analysis was not performed because the number of studies was too few to detect an asymmetric funnel [30]. All analyses were performed using Comprehensive Meta-Analysis Statistical Software, version 2.0 (Biostat, Englewood, NJ, USA).

## Results

### Literature search

**Fig 1:**
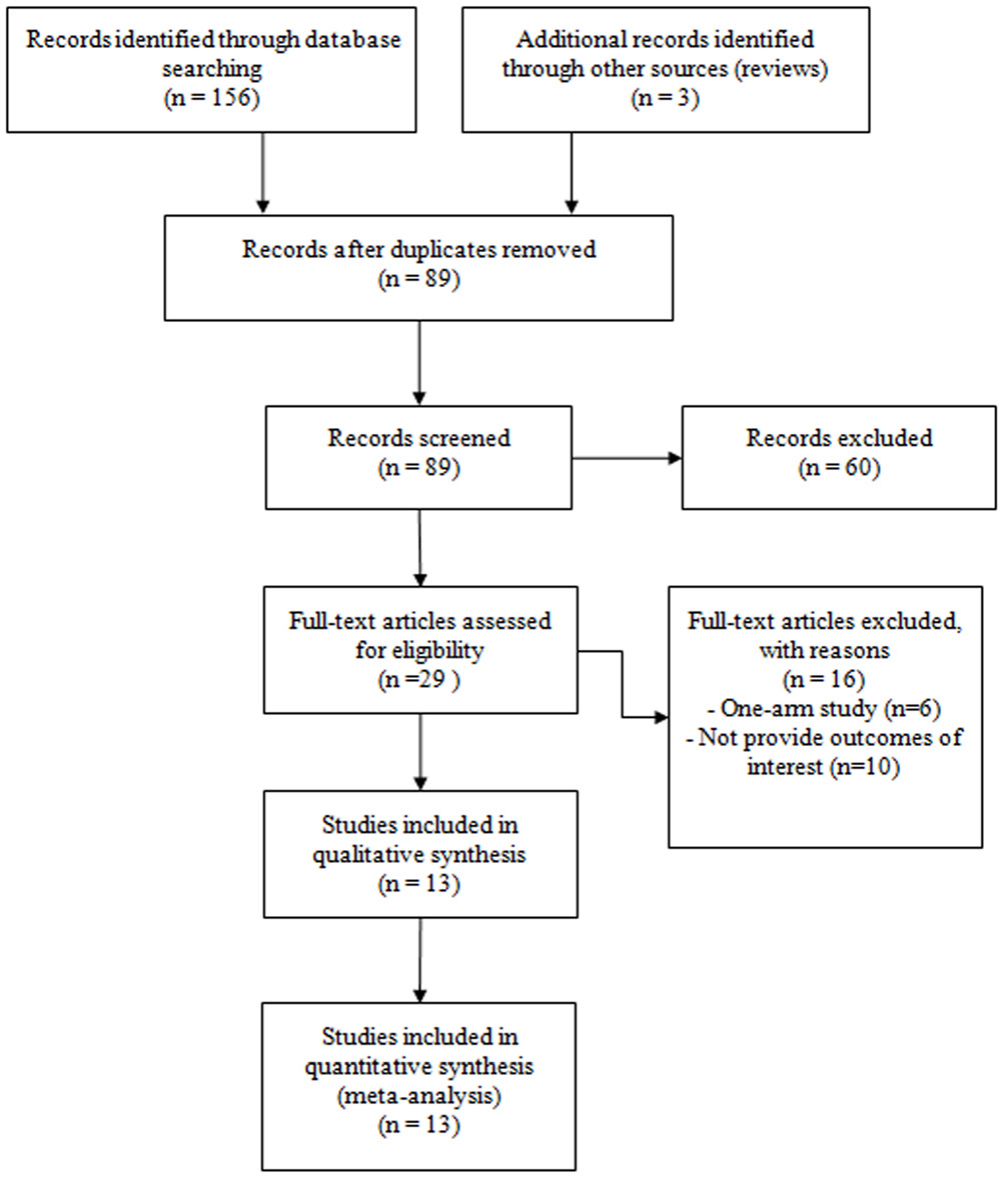
Flow chart for study selection.

A total of 89 articles were assessed for eligibility based on their abstracts and our inclusion/exclusion criteria, and 60 were excluded. After full text review of the remaining 29 studies, 16 studies were excluded due to lack of outcomes of interest (n = 10) or for being one-arm studies (n = 6), as shown in Fig 1.

The remaining 13 articles [11–18,31–35] evaluating patients with primary brain tumors or brain metastasis for recurrent tumor vs. necrosis using MR perfusion or MR spectroscopy were used for the meta-analysis. The characteristics of all 13 studies are summarized in Table 1.

**Table 1:** Baseline characteristics of the 13 studies included in the meta-analysis.

First Author	Study Design	Type of tumor	WHO Grade	Differentiating (Tumor vs. Necrosis) Method	MRI type	Group	Patients No.	Age (yrs)	Male (%)
Prager (2015)[35]	Retrospective	Primary glioblastoma or anaplastic astrocytoma	NA	Histopathology at repeat surgical excision	1.5T & 3T	Recurrent tumor	58	55	75%
						Necrosis	10		
Alexiou (2014)[31]	Prospective	GBM in 27, anaplastic astrocytoma in two and one anaplastic oligodendroglioma.	NA	Surgical excision in 2 cases, remaining cases on “wait-and-see” prospective clinical & imaging follow-up	1.5-T	Recurrent tumor	24	62	70%
						Necrosis	6		
Di Costanzo (2014)[32]	Prospective	Gliomas	Grade IV	Imaging & clinical evidences (enhancement or mass effect in tumors vs. regression or stable-appearing in necrosis) in four follow-up examinations.	3.0T	Recurrent tumor	21	63	62%
						Necrosis	8		
D'Souza (2014)[33]	Prospective	Gliomas	Grade III & IV	Histological analysis on surgery or biopsy (n = 22), or by clinical follow-up with serial MRI +/- PET for at least 6 months.	3.0T	Recurrent tumor	19	37	84%
						Necrosis	10	47	50%
Shin (2014)[34]	Retrospective	Gliomas	Grade II, III & IV	16/19 recurrent tumor and 8/12 treatment-necrosis were confirmed by histopathology, rest were confirmed by clinical radiological follow-up of 8–18 months.	3.0T	Recurrent tumor	19	54.5	55%
						Necrosis	12		
Huang (2011)[12]	Retrospective	Metastatic brain tumor	NA	Histopathological confirmation	1.5T	Recurrent tumor	23	56	35%
				(n = 4) from surgical resection, or radiological progression pattern combined with clinical follow-up (n = 29).					
						Necrosis	10	63	50%
Xu (2011)[11]	Prospective	Primary brain tumors	Grade II: 4, grade III:14 and grade IV: 17	Histopathology in 23 cases (18 from biopsy, 5 from resection), and clinical + serial radiological follow-up in 13 cases (6–31 months).	3.0T	Recurrent tumor	20	45	54%
						Necrosis	15		
Matsusue (2010)[14]	Retrospective	Primary brain tumors	Grade II: 9, grade III:2 and grade IV: 4	Histopathology in 3 cases, and combined clinical & serial MR follow-up (up to 15 months) in 12 cases.	3.0T	Recurrent tumor	10	50	50%
						Necrosis	5	43	80%
Mitsuya (2010)[13]	Prospective	Metastatic brain tumor	NA	Clinical and serial MR follow-up every 1–3 months.	1.5T	Recurrent tumor	7	53	43%
						Necrosis	21	62	57%
Barajas (2009)[15]	Retrospective	Metastatic brain tumor	NA	Either confirmed histologically or clinicoradiologically, with serial MR follow-up every 1–3 months.	1.5T	Recurrent tumor	27	NA	41%
						Necrosis			
Weybright (2005)[16]	Retrospective	Intracranial neoplasm	Grades II—IV: 24	Confirmed either by histo-pathology from biopsies, surgical resections or autopsy, or by serial MR + clinical exam.	1.5T	Recurrent tumor	16	34	55%
						Necrosis	12		
Rock (2002)[17]	Retrospective	Malignant gliomas	NNA	Histopathology from biopsies or surgical resected specimens.	1.5T	Recurrent tumor	18	18+	NA
						Necrosis	15		
Kamada (1997)[18]	Prospective	Primary brain tumors	Grade II: 1, grade III: 3 and grade IV: 6	All except one were confirmed by histology from surgical resected specimens, one radiation necrosis patient was conformed by clinical + imaging evidences	1.5T	Recurrent tumor	6	39	NA
						Necrosis	5	53	

Abbreviations: NA, not available; WHO, World Health Organization; MRI, magnetic resonance imaging; yrs, years.

### Study characteristics and clinical outcomes

As shown in Table 1, six studies [11,13,18,31–33] were prospective and seven [12,14–17,34,35] were retrospective two-armed studies. The 397 patients encompassed by the 13 studies (Table 1) had an average age ranging from 34 years to 63 years and the majority of patients were male. The majority of patients also had recurrent tumor rather than radiation necrosis. Of the seven studies [11,14,16,18,32–34] which provided WHO grades, most tumors were grades II-IV.

The outcomes of all included studies are summarized in Table 2. Ten studies [11–15,31–35] evaluated the efficacy of using MR perfusion to differentiate recurrent tumor vs. necrosis, six studies used MR spectroscopy [12,14,16, 18,32,33], and only four studies [12, 14,32,33] used both MR spectroscopy and MR perfusion to differentiate radiation necrosis from tumor recurrence.

**Table 2:** Summary of the functional outcomes among studies selected for meta-analysis.

First Author	Relative cerebral blood volume	Ratio of Cho/Cr	Ratio of Cho/NAA
	(Recurrent tumor vs. Necrosis)	(Recurrent tumor vs. Necrosis)	(Recurrent tumor vs. Necrosis)
Prager (2015)[35]	1.81 (1.46, 2.58) vs. 1.015 (0.82, 1.46) †	NA	NA
Alexiou (2014)[31]	6.71 (0.41) vs. 1.68 (0.42)	NA	NA
Di Costanzo (2014)[32]	1.73 (0.56) vs. 0.86 (0.37)	2.12 (0.64) vs. 1.90 (0.32)	2.84 (1.40) vs. 1.69 (0.48)
D'Souza (2014)[33]	3.01 (1.82) vs. 0.85 (0.34)	2.27 (0.59) vs. 1.26 (0.50)	NA
Shin (2014)[34]	4.40 (3.07) vs. 2.08 (1.15)	NA	NA
Huang (2011)[12]	2.49 (1.73) vs. 1.03 (0.23)	1.72 (1.10) vs. 1.34 (0.48)	1.32 (1.25) vs. 1.18 (0.37)
Xu (2011)[11]	4.36 (1.98) vs. 1.28 (0.64)	NA	NA
Matsusue (2010)[14]	3.33 (1.16) vs. 1.82 (0.80)	1.87 (0.39) vs. 1.11 (0.66)	1.56 (0.82) vs. 1.16 (0.91)
Mitsuya (2010)[13]	3.5 (2.1–10)* vs. 1.0 (0.39–2.57)*	NA	NA
Weybright (2005)[15]	NA	2.52 (1.66–4.26)^ vs. 1.57 (0.72–1.76)^	3.48 (1.70–6.47)^ vs. 1.31 (0.83–1.78)^
Rock (2002)[16]	NA	1.79 (0.79) vs. 0.89 (1.04)	NA
Barajas (2009)[17]	2.38 (0.95) vs. 1.54 (0.92)	NA	NA
Kamada (1997)[18]	NA	3.07 (0.23) vs. 2.07 (0.72)	NA

Data are expressed as mean (standard deviation) unless otherwise stated.

* Data expressed as median (range).

^†^ Data expressed as median (IQR).

^ Data expressed as mean (range).

Abbreviations: Cho, choline; Cr, creatine; NAA, N-acetyl aspartate; NA, not available.

### Difference of rCBV in tumor vs. necrosis

**Fig 2:**
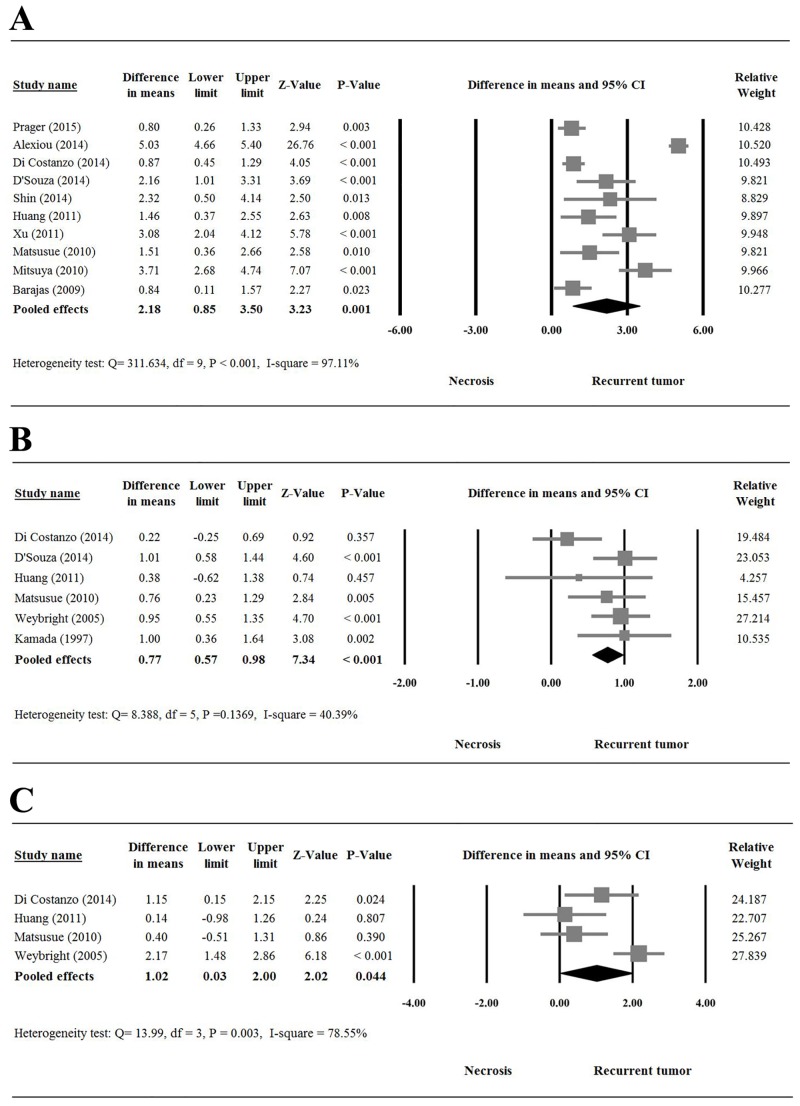
Forest plots showing results of the meta-analysis regarding (A) relative cerebral blood volume (rCBV), (B) Cho/Cr ratios, and (C) Cho/NAA ratios for recurrent tumor vs. radiation necrosis groups.

Fig 2A is a forest plot of the difference in means of rCBV. Ten [11–15, 31–35] of the 13 studies provided complete numerical data evaluating rCBV for recurrent tumor vs. necrosis, and were included in the meta-analysis. There was evidence of heterogeneity regarding the rCBV values among the 10 studies (Q statistic = 311.634, I^2^ = 97.11%, P < 0.001); therefore, a random-effects model of analysis was used. Pooled differences in means (2.18, 95%CI = 0.85 to 3.50) indicated the mean rCBV in a contrast-enhancing lesion was significantly higher in tumor recurrence compared with radiation injury (P = 0.001, Fig 2A).

### Ratios of Cho/Cr and Cho/NAA in tumor vs. necrosis

Fig 2B is a forest plot of the difference in means of the Cho/Cr ratio. Six [12, 14, 16, 18,32,33] of the 13 studies showed no evidence of heterogeneity regarding Cho/Cr ratio evaluation and were included in the meta-analysis using a fixed-effect model of analysis (Q statistic = 8.388, I^2^ = 40.39%, P = 0.137). The pooled difference in means (0.77, 95%CI = 0.57 to 0.98) indicated the mean Cho/Cr ratio was significantly higher in tumor recurrence than in necrosis (P = 0.000, Fig 2B).

Fig 2C is a forest plot of the difference in means of the Cho/NAA ratio. Four [12, 14, 16,32] of the 13 studies provided completed numerical data regarding Cho/NAA ratio for recurrent tumor vs. necrosis, and were included in the meta-analysis. There was evidence of heterogeneity regarding the Cho/NAA ratio among the four studies (Q statistic = 13.99, I^2^ = 78.55%, P = 0.003); therefore, a random-effects model of analysis was used. There was significant difference in Cho/NAA ratio between recurrent tumor and necrosis (1.02, 95%CI = 0.03 to 2.00, P = 0.044, Fig 2C).

### Sensitivity analysis

Sensitivity analysis was performed using the leave-one-out approach in which the meta-analysis of the rCBV and ratios of Cho/Cr and Cho/NAA were performed with each study removed in turn (Table 3). For rCBV and ratio of Cho/Cr, the direction and magnitude of combined estimates did not vary markedly with the removal of the studies, indicating the data was not overly influenced by each study. The four pooled differences in means of Cho/NAA ratios remained significant after each study was removed, in turn, except the removal of Di Costanzo (2014)[32] which changed it to non-significant, indicating Di Costanzo (2014) might influenced the pooled estimate.

**Table 3:** Sensitivity-analysis for recurrent tumor vs. radiation necrosis groups.

First author (year)	Statistics with study removed				
	Points	Lower limit	Upper limit	Z-Value	P-Value
**Relative cerebral blood volume (rCBV)**					
Prager (2015)	2.34	0.90	3.78	3.18	0.001
Alexiou (2014)	1.77	1.10	2.43	5.21	0.000
Di Costanzo (2014)	2.33	0.89	3.76	3.18	0.001
D'Souza (2014)	2.18	0.75	3.60	2.99	0.003
Shin (2014)	2.16	0.76	3.56	3.03	0.002
Huang (2011)	2.25	0.83	3.68	3.10	0.002
Xu (2011)	2.08	0.64	3.51	2.84	0.005
Matsusue (2010)	2.25	0.83	3.67	3.10	0.002
Mitsuya (2010)	2.01	0.58	3.43	2.76	0.006
Barajas (2009)	2.33	0.90	3.76	3.19	0.001
**Cho/Cr ratios**					
Di Costanzo (2014)	0.91	0.68	1.14	7.73	0.000
D'Souza (2014)	0.70	0.47	0.94	5.85	0.000
Huang (2011)	0.79	0.58	1.00	7.34	0.000
Matsusue (2010)	0.78	0.55	1.00	6.77	0.000
Weybright (2005)	0.71	0.47	0.95	5.73	0.000
Kamada (1997)	0.75	0.53	0.96	6.70	0.000
**Cho/NAA ratios**					
Di Costanzo (2014)	0.95	-0.41	2.32	1.37	0.172
Huang (2011)	1.27	0.18	2.37	2.29	0.022
Matsusue (2010)	1.22	0.04	2.40	2.02	0.043
Weybright (2005)	0.58	0.00	1.16	1.97	0.049

Abbreviations: Cho, choline; Cr, creatine; NAA, N-acetyl aspartate.

### Publication bias

Publication bias regarding outcomes was not assessed because there were fewer than ten studies required to detect funnel plot asymmetry [30].

## Discussion

The aim of our study was to evaluate the diagnostic effectiveness of MR perfusion and MR spectroscopy in differentiating recurrent tumor from radiation necrosis. Our meta-analysis showed that both average rCBV and average Cho/Cr and Cho/NAA ratios were significantly higher in tumor recurrence compared with radiation injury (all P < 0.05). We performed sensitivity analysis and tested for homogeneity as part of our study. A χ^2^-based test of homogeneity was performed using Cochran’s Q statistic and I^2^. The studies which used Cho/Cr ratio to distinguish tumor recurrence from necrosis showed good homogeneity. We also tested for reliability based on sensitivity analysis. Sensitivity analysis using leave-one-out approach evaluated the influence of each study on the pooled estimate for both functional scores. The direction and magnitude of the combined estimates did not change markedly with the exclusion of individual studies, indicating that our meta-analysis had good reliability.

Compared with other studies, our meta-analysis contributed to the collective knowledge of MR spectroscopy and MR perfusion’s versatile roles. As our study was not restricted to patients with a specific type of primary brain tumor, patients with brain metastasis were included as well (since the number of studies in any one category was insufficient for a meta-analysis) which added clinical usefulness to our findings.

Several other studies evaluating the use of either MR spectroscopy or MR perfusion also found rCBV [7,36–39], Cho/Cr and Cho/NAA ratios [3,40–45] to be good predictors of recurrent tumor, which is consistent with our findings. Most of these studies were single-arm and therefore were not included in our meta-analysis. Elias et al. [44] found that Cho/NAA and NAA/Cr ratios were best able to discriminate tumor recurrence from treatment necrosis. Similar to our findings, Guo et al. [45] showed that in high grade gliomas, higher Cho/NAA ratios were associated with a greater probability of tumor infiltration. Smith et al. also found that an elevated Cho/NAA ratio correlated with tumor recurrence [41].

The role of N-acetyl aspartate (NAA) in discriminating tumor recurrence from necrosis is more controversial. In our study, a significant difference in the ratios of Cho/NAA between the two groups was noted. However, when analyzing MR spectra of suspicious lesions, Krouwer et al. found increased Cho/NAA ratios not only in tumor but also in nonneoplastic brain lesions whose histology revealed inflammation and reactive astrogliosis [46,47]. Small sample sizes may explain some of these inconsistencies across studies with regards to Cho/NAA ratios. The discrepancy in results based on Cho/NAA ratios may be also related to time after treatment, particularly in cases of tumor necrosis. For example, choline has been reported to increase during the first few months after radiation therapy and then decrease as treatment necrosis begins to appear [3,10,48]. Treatment necrosis has also been reported to show variable changes in choline and creatine intensities over time [3,10,19,42, 48–51]. Estève et al. [48] observed a significant decrease in NAA/Cho in normal brain tissue 4 months after irradiation. Thus, high Cho/NAA ratios may be due to early radiation-induced inflammation, demyelination, or gliosis which can decrease over time.

Our study had several limitations including the limited number of studies available for the meta-analysis. In addition, the operators/observers who evaluated rCBV and other MR spectroscopy data might not be blinded to other clinical data. The MR spectroscopy parameters used across different studies were not consistent, and different studies used different cut-off values of metabolites for comparison. Future studies using multi-voxel spectroscopy may be needed to determine cut-off values for metabolite ratios. Delayed radiation effects can have a long latency period, as already discussed, and may skew MR spectroscopy results. The sensitivity of perfusion imaging to artifacts is another limitation. Finally, there may have been a selection bias with regards to the studies chosen for the meta-analysis.

In conclusion, based on the results of our meta-analysis, rCBV and ratios of Cho/Cr and Cho/NAA were higher in recurrent tumors than in radiation necrosis. MR spectroscopy using Cho/NAA and Cho/Cr ratios and MR perfusion using rCBV may increase the accuracy of differentiating necrosis from recurrent tumor in patients with primary brain tumors or metastases.

## Supporting Information

S1 File A sample of abstract review used in the search strategy. (DOC)

S1 PRISMA Checklist Preferred Reporting Items for Systematic Reviews and Meta-Analyses 2009 Checklist. (DOC)
