# Supplementary material for: Differentiating Radiation-Induced Necrosis from Recurrent Brain Tumor Using MR Perfusion and Spectroscopy: A Meta-Analysis
Source: PLoS One. 2016 Jan 7;11(1):e0141438. doi: 10.1371/journal.pone.0141438 (PMC4712150; doi:10.1371/journal.pone.0141438)
Supplement: S1 File — (DOC) [file pone.0141438.s001.doc]

**Abstract Review Report**

The articles about “differentiating radiation-induced necrosis from recurrent brain tumor using MR perfusion and spectroscopy” were searched in database including PubMed, Cochrane and Embase databases. Results are summarized a table below. All 156 records identified were screened and after removal of the identical records, there are 8 qualified publications.

**Search Strategy**

Other search filters: Not letters, editorials, comments, reviews or case reports.

| Database | Key words and the combination | Articles found (N) | Articles excluded (N) | Qualified articles (N) |
| --- | --- | --- | --- | --- |
| PubMed* | (magnetic resonance spectroscopy OR MR spectroscopy OR magnetic resonance perfusion OR MR) AND (brain tumors OR glioma) AND (recurrence OR recurrent) AND (radiation necrosis OR radiation injury) | 97 | 84 | 13 |
| Cochrane** | (magnetic resonance spectroscopy OR MR spectroscopy OR magnetic resonance perfusion OR MR) AND (brain tumors OR glioma) AND (recurrence OR recurrent) | 9 | 9 | 0 |
| Embase *** | (magnetic resonance spectroscopy OR MR spectroscopy OR magnetic resonance perfusion OR MR) AND (brain tumors OR glioma) AND (recurrence OR recurrent) AND (radiation necrosis OR radiation injury) | 50 | 47 | 3 |
| Hand search from other sources |  |  |  | 13 |
| **Total number** | | **156** | **127** | **29** |

* PubMed search filters: Abstract available, Humans, English.

** Cochrane: Human, Abstract available, English.

*** Embase search filters: Article, Human, Clinical article, English.

**List of all the qualified articles**

1. Chung WJ, Kim HS, Kim N, Choi CG, Kim SJ. Recurrent glioblastoma: optimum area under the curve method derived from dynamic contrast-enhanced T1-weighted perfusion MR imaging. Radiology. 2013;269(2): 561-568.
2. Reddy K, Westerly D, Chen C. MRI patterns of T1 enhancing radiation necrosis versus tumour recurrence in high-grade gliomas. J Med Imaging Radiat Oncol. 2013;57(3): 349-355.
3. Xu JL, Shi DP, Dou SW, Li YL, Yan FS. Distinction between postoperative recurrent glioma and delayed radiation injury using MR perfusion weighted imaging. J Med Imaging Radiat Oncol. 2011;55(6): 587-594.
4. Elias AE, Carlos RC, Smith EA, Frechtling D, George B, Maly P, et al. MR spectroscopy using normalized and non-normalized metabolite ratios for differentiating recurrent brain tumor from radiation injury. Acad Radiol. 2011;18(9): 1101-1108.
5. Huang J, Wang AM, Shetty A, Maitz AH, Yan D, Doyle D, et al. Differentiation between intra-axial metastatic tumor progression and radiation injury following fractionated radiation therapy or stereotactic radiosurgery using MR spectroscopy, perfusion MR imaging or volume progression modeling. Magn Reson Imaging. 2011;29(7): 993-1001.
6. Bobek-Billewicz B, Stasik-Pres G, Majchrzak H, Zarudzki L. Differentiation between brain tumor recurrence and radiation injury using perfusion, diffusion-weighted imaging and MR spectroscopy. Folia Neuropathol. 2010;48(2): 81-92.
7. Mitsuya K, Nakasu Y, Horiguchi S, Harada H, Nishimura T, Bando E, et al. Perfusion weighted magnetic resonance imaging to distinguish the recurrence of metastatic brain tumors from radiation necrosis after stereotactic radiosurgery. J Neurooncol. 2010;99(1): 81-88.
8. Matsusue E, Fink JR, Rockhill JK, Ogawa T, Maravilla KR. Distinction between glioma progression and post-radiation change by combined physiologic MR imaging. Neuroradiology. 2010;52(4): 297-306.
9. Barajas RF, Chang JS, Sneed PK, Segal MR, McDermott MW, Cha S, et al. Distinguishing recurrent intra-axial metastatic tumor from radiation necrosis following gamma knife radiosurgery using dynamic susceptibility-weighted contrast-enhanced perfusion MR imaging. AJNR Am J Neuroradiol. 2009;30(2): 367-372.
10. Di Costanzo A, Trojsi F, Giannatempo GM, Vuolo L, Popolizio T, Catapano D, et al. Spectroscopic, diffusion and perfusion magnetic resonance imaging at 3.0 Tesla in the delineation of glioblastomas: preliminary results. J Exp Clin Cancer Res. 2006;25(3): 383-390.
11. Weybright P, Sundgren PC, Maly P, Hassan DG, Nan B, Rohrer S, et al. Differentiation between brain tumor recurrence and radiation injury using MR spectroscopy. AJR Am J Roentgenol. 2005;185(6): 1471-1476.
12. Rabinov JD, Lee PL, Barker FG, Louis DN, Harsh GR, Cosgrove GR, et al. In vivo 3-T MR spectroscopy in the distinction of recurrent glioma versus radiation effects: initial experience. Radiology. 2002;225(3): 871-879.
13. Rock JP, Hearshen D, Scarpace L, Croteau D, Gutierrez J, Fisher JL, et al. Correlations between magnetic resonance spectroscopy and image-guided histopathology, with special attention to radiation necrosis. Neurosurgery. 2002;51(4): 912-919.
14. Lai PH, Ho JT, Chen WL, Hsu SS, Wang JS, Pan HB, et al. Brain abscess and necrotic brain tumor: discrimination with proton MR spectroscopy and diffusion-weighted imaging. AJNR Am J Neuroradiol. 2002;23(8): 1369-1377.
15. Kamada K, Houkin K, Abe H, Sawamura Y, Kashiwaba T. Differentiation of cerebral radiation necrosis from tumor recurrence by proton magnetic resonance spectroscopy. Neurol Med Chir (Tokyo). 1997;37(3): 250-256.
16. Taylor JS, Langston JW, Reddick WE, Kingsley PB, Ogg RJ, Pui MH, et al. Clinical value of proton magnetic resonance spectroscopy for differentiating recurrent or residual brain tumor from delayed cerebral necrosis. Int J Radiat Oncol Biol Phys. 1996;36(5): 1251-1261.
17. Ikehira H, Miyamoto T, Yasukawa T, Obata T, Katoh H, Koga M, et al. Differences in metabolic and morphological reactions after radiation therapy: proton NMR spectroscopy and imaging of patients with intracranial tumors. Radiat Med. 1995;13(5): 199-204.
18. Shah R, Vattoth S, Jacob R, Manzil FF, O'Malley JP, Borghei P, et al. Radiation necrosis in the brain: imaging features and differentiation from tumor recurrence. Radiographics. 2012;32(5): 1343-1359.
19. Narang J, Jain R, Arbab AS, Mikkelsen T, Scarpace L, Rosenblum ML, et al. Differentiating treatment-induced necrosis from recurrent/progressive brain tumor using nonmodel-based semiquantitative indices derived from dynamic contrast-enhanced T1-weighted MR perfusion. Neuro Oncol 2011;13(9): 1037-1046.
20. Amin A, Moustafa H, Ahmed E, El-Toukhy M. Glioma residual or recurrence versus radiation necrosis: accuracy of pentavalent technetium-99m-dimercaptosuccinic acid [Tc-99m (V) DMSA] brain SPECT compared to proton magnetic resonance spectroscopy (1H-MRS): initial results. J Neurooncol. 2012;106(3): 579-587.
21. Nakajima T, Kumabe T, Kanamori M, Saito R, Tashiro M, Watanabe M, et al. Differential diagnosis between radiation necrosis and glioma progression using sequential proton magnetic resonance spectroscopy and methionine positron emission tomography. Neurol Med Chir (Tokyo). 2009;49(9): 394-401.
22. Prager AJ, Martinez N, Beal K, Omuro A, Zhang Z, Young RJ. Diffusion and perfusion MRI to differentiate treatment-related changes including pseudoprogression from recurrent tumors in high-grade gliomas with histopathologic evidence. AJNR Am J Neuroradiol. 2015;36(5):877-85.
23. Boxerman JL, Ellingson BM, Jeyapalan S, Elinzano H, Harris RJ, Rogg JM, et al. Longitudinal DSC-MRI for distinguishing tumor recurrence from pseudoprogression in patients with a high-grade glioma. Am J Clin Oncol. 2014 Nov 26. [Epub ahead of print].
24. Kim DY, Kim HS, Goh MJ, Choi CG, Kim SJ. Utility of intravoxel incoherent motion MR imaging for distinguishing recurrent metastatic tumor from treatment effect following gamma knife radiosurgery: initial experience. AJNR Am J Neuroradiol. 2014;35(11): 2082-2090.
25. Shin KE, Ahn KJ, Choi HS, Jung SL, Kim BS, Jeon SS, et al. DCE and DSC MR perfusion imaging in the differentiation of recurrent tumour from treatment-related changes in patients with glioma. Clin Radiol. 2014;69(6): e264-e272
26. Di Costanzo A, Scarabino T, Trojsi F, Popolizio T, Bonavita S, de Cristofaro M, et al. Recurrent glioblastoma multiforme versus radiation injury: a multiparametric 3-T MR approach. Radiol Med. 2014;119(8): 616-624.
27. Alexiou GA, Zikou A, Tsiouris S, Goussia A, Kosta P, Papadopoulos A, et al. Comparison of diffusion tensor, dynamic susceptibility contrast MRI and (99m)Tc-Tetrofosmin brain SPECT for the detection of recurrent high-grade glioma. Magn Reson Imaging. 2014;32(7):854-859.
28. Sherif M.F., Salem F.M., Almahallawy M.A., Algawad A.M.A., Hammad Q.M.

[Role of magnetic resonance spectroscopy in differentiation between recurrence of glioma and post radiation injury](http://www.embase.com.ezp-prod1.hul.harvard.edu/search/results?subaction=viewrecord&rid=5&page=2&id=L602175378). Egyptian J Radiol Nucl Med. 2014;45(4):1233-1240.

1. D'Souza MM, Sharma R, Jaimini A, Panwar P, Saw S, Kaur P, et al. 11C-MET PET/CT and advanced MRI in the evaluation of tumor recurrence in high-grade gliomas. Clin Nucl Med. 2014;39(9): 791-798.

**Articles of Interest (several abstracts listed below as samples used in search strategy)**

#1. Prager AJ, Martinez N, Beal K, Omuro A, Zhang Z, Young RJ. Diffusion and perfusion MRI to differentiate treatment-related changes including pseudoprogression from recurrent tumors in high-grade gliomas with histopathologic evidence. AJNR Am J Neuroradiol. 2015;36(5):877-85.

| 1st author | Year | Type of study | Patient number | Type of patients |
| --- | --- | --- | --- | --- |
| Prager AJ | 2015 | Clinical study | 68 | glioma |
| Intervention | We retrospectively identified 68 consecutive patients with high-grade gliomas treated by surgical resection followed by radiation therapy and temozolomide, who then developed increasing enhancing mass lesions indeterminate for treatment-related changes versus recurrent tumor. All lesions were diagnosed by histopathology at repeat surgical resection. ROI analysis was performed of the enhancing lesion on the ADC and DSC maps. Measurements made by a 2D ROI of the enhancing lesion on a single slice were recorded as ADC_Lesion_ and rCBV_Lesion_, and measurements made by the most abnormal small fixed diameter ROI as ADC_ROI_ and rCBV_ROI_. Statistical analysis was performed with Wilcoxon rank sum tests with P = .05. | | | |
| Outcome | Ten of the 68 patients (14.7%) had treatment-related changes, while 58 patients (85.3%) had**recurrent**tumor only (n = 19) or**recurrent**tumor mixed with treatment effect (n = 39). DWI analysis showed higher ADC_Lesion_ in treatment-related changes than in**recurrent**tumor (P = .003). DSC analysis revealed lower relative cerebral blood volume (rCBV)_Lesion_ and rCBV_ROI_ in treatment-related changes (P = .003 and P = .011, respectively). Subanalysis of patients with suspected pseudoprogression also revealed higher ADC_Lesion_ (P = .001) and lower rCBV_Lesion_ (P = .028) and rCBV_ROI_ (P = .032) in treatment-related changes. Applying a combined ADC_Lesion_ and rCBV_Lesion_ model did not outperform either the ADC or rCBV metric alone. | | | |
| Conclusion | Treatment-related changes showed higher diffusion and lower**perfusion**than**recurrent**tumor. Similar correlations were found for patients with suspected pseudoprogression. | | | |

#2. Boxerman JL, Ellingson BM, Jeyapalan S, Elinzano H, Harris RJ, Rogg JM, et al. Longitudinal DSC-MRI for distinguishing tumor recurrence from pseudoprogression in patients with a high-grade glioma. Am J Clin Oncol. 2014 Nov 26. [Epub ahead of print].

| 1st author | Year | Type of study | Patient number | Type of patients |
| --- | --- | --- | --- | --- |
| Boxerman JL | 2014 | Clinical study | 19 | glioma |
| Intervention | A subset of patients with high-grade glioma on a phase II clinical trial with temozolomide, paclitaxel poliglumex, and concurrent radiation were assessed. Nine patients (3 grade III, 6 grade IV), with a total of 19 enhancing lesions demonstrating progressive enhancement (≥25% increase from nadir) on postchemoradiation conventional contrast-enhanced MRI, had serial DSC-MRI. Mean leakage-corrected rCBV within enhancing lesions was computed for all postchemoradiation time points. | | | |
| Outcome | Of the 19 progressively enhancing lesions, 10 were classified as PsP and 9 as PD by biopsy/surgery or serial enhancement patterns during interval follow-up MRI. Mean rCBV at initial progressive enhancement did not differ significantly between PsP and PD (2.35 vs. 2.17; P=0.67). However, change in rCBV at first subsequent follow-up (-0.84 vs. 0.84; P=0.001) and the overall linear trend in rCBV after initial progressive enhancement (negative vs. positive slope; P=0.04) differed significantly between PsP and PD. | | | |
| Conclusion | Longitudinal trends in rCBV may be more useful than absolute rCBV in distinguishing PsP from PD in chemoradiation-treated high-grade gliomas with DSC-MRI. Further studies of DSC-MRI in high-grade**glioma**as a potential technique for distinguishing PsP from PD are indicated. | | | |

#3. Kim DY, Kim HS, Goh MJ, Choi CG, Kim SJ. Utility of intravoxel incoherent motion MR imaging for distinguishing recurrent metastatic tumor from treatment effect following gamma knife radiosurgery: initial experience. AJNR Am J Neuroradiol. 2014;35(11): 2082-2090.

| 1st author | Year | Type of study | Patient number | Type of patients |
| --- | --- | --- | --- | --- |
| Kim DY | 2014 | Clinical study | 91 | Brain tumor |
| Intervention | Ninety-one consecutive patients with metastatic brain tumor treated with gamma knife radiosurgery were assessed by using intravoxel incoherent motion imaging. Two readers independently calculated the 90th percentile and the 10th percentile histogram cutoffs for perfusion, normalized CBV, diffusion, and ADC. Areas under the receiver operating characteristic curve and interreader agreement were assessed. | | | |
| Outcome | With the combination of the 90th percentile histogram cutoff for**perfusion**and the 10th percentile histogram cutoff for diffusion, the sensitivity and specificity for differentiating**recurrent**tumor and treatment were 79.5% and 92.3% for reader 1 and 84.6% and 94.2% for reader 2, respectively. With the combination of the 90th percentile histogram cutoff for normalized CBV and the 10th percentile histogram cutoff for ADC, the sensitivity and specificity for differentiating**recurrent**tumor and treatment were 69.2% and 100.0% for reader 1 and 74.3% and 100.0% for reader 2, respectively. Compared with the combination of 90th percentile histogram cutoff for normalized CBV and the 10th percentile histogram cutoff for ADC, adding intravoxel incoherent motion to 90th percentile histogram cutoff for normalized CBV substantially improved the diagnostic accuracy for differentiating**recurrent**tumor and treatment from 86.8% to 92.3% for reader 1 and from 89.0% to 93.4% for reader 2, respectively. The intraclass correlation coefficients between readers were higher for**perfusion**parameters (intraclass correlation coefficient range, 0.84-0.89) than for diffusion parameters (intraclass correlation coefficient range, 0.68-0.79). | | | |
| Conclusion | Following gamma knife radiosurgery, intravoxel incoherent motion**MR**imaging can be used as a noninvasive imaging biomarker for differentiating**recurrent**tumor from treatment effect in patients with metastatic**brain**tumor. | | | |

#4. Shin KE, Ahn KJ, Choi HS, Jung SL, Kim BS, Jeon SS, et al. DCE and DSC MR perfusion imaging in the differentiation of recurrent tumour from treatment-related changes in patients with glioma. Clin Radiol. 2014;69(6): e264-e272

| 1st author | Year | Type of study | Patient number | Type of patients |
| --- | --- | --- | --- | --- |
| Shin KE | 2014 | Clinical study | 31 | glioma |
| Intervention | Thirty-one patients with histologically diagnosed gliomas and increased enhancement after or during concurrent (chemo-) radiation therapy were enrolled. They underwent dynamic contrast-enhanced (DCE) permeability MRI followed by dynamic susceptibility contrast (DSC) perfusion MRI. The vascular transfer constant (rK(trans)) and initial areas under the concentration curve (riAUC) were obtained from DCE MRI, and cerebral blood volume (rCBV) was obtained from DSC MRI. Patients were classified as having treatment-related changes or recurrent tumours based on clinicoradiological results or pathological results from surgery. | | | |
| Outcome | Nineteen patients were diagnosed as having recurrences and 12 patients as having treatment-related changes. The rK(trans), riAUC, and rCBV values in the**recurrent**group were significantly higher than the values in the group with treatment-related changes (p < 0.05). For all 31 patients, there was no significant difference between DSC MRI and DCE MRI for the differentiating power between**recurrence**and treatment-related changes (p = 0.7227). However, when including only the 24 patients with concordant values of rK(trans) and riAUC, DCE MRI showed a significant AUC value of 0.786 in the receiver operating characteristic (ROC) curve analysis (p = 0.003), whereas DSC MRI did not (AUC = 0.643, p = 0.229). | | | |
| Conclusion | MRI**perfusion**images appear to show promise in distinguishing treatment-related changes from**recurrent**tumours. When both rK(trans) and riAUC show concordant values, DCE MRI seems to be more powerful than DSC MRI in the differentiation of**recurrence**from treatment-related changes. | | | |

#5. Di Costanzo A, Scarabino T, Trojsi F, Popolizio T, Bonavita S, de Cristofaro M, et al. Recurrent glioblastoma multiforme versus radiation injury: a multiparametric 3-T MR approach. Radiol Med. 2014;119(8): 616-624.

| 1st author | Year | Type of study | Patient number | Type of patients |
| --- | --- | --- | --- | --- |
| Di Costanzo A | 2014 | Clinical study | 21 | glioma |
| Intervention | Twenty-nine patients with histologically verified high-grade gliomas, who had undergone surgical resection and radiotherapy, and had developed new contrast-enhancing lesions close to the treated tumour, underwent MRI, (1)H-MRSI, DWI and PWI at regular time intervals. The metabolite ratios choline (Cho)/normal( n )Cho n , N-acetylaspartate (NAA)/NAA n , creatine (Cr)/Cr n , lactate/lipids (LL)/LL n , Cho/Cr n , NAA/Cr n , Cho/NAA, NAA/Cr and Cho/Cr were derived from (1)H-MRSI; the apparent diffusion coefficient (ADC) from DWI; and the relative cerebral blood volume (rCBV) from PWI. | | | |
| Outcome | In serial MRI,**recurrent**gliomas showed a progressive enlargement, and radiation injuries showed regression or no modification. Discriminant analysis showed that discrimination accuracy was 79.3 % when considering only the metabolite ratios (predictor, Cho/Cr n ), 86.2 % when considering ratios and ADC (predictors, Cho/Cr n and ADC), 89.7 % when considering ratios and rCBV (predictors, Cho/Cr n , Cho/Cr and rCBV), and 96.6 % when considering ratios, ADC and rCBV (predictors, Cho/Cho n , ADC and rCBV). | | | |
| Conclusion | The multiparametric 3-T**MR**assessment based on (1)H-MRSI, DWI and PWI in addition to MRI is a useful tool to discriminate tumour**recurrence**/progression from radiation effects. | | | |

#6. Alexiou GA, Zikou A, Tsiouris S, Goussia A, Kosta P, Papadopoulos A, et al. Comparison of diffusion tensor, dynamic susceptibility contrast MRI and (99m)Tc-Tetrofosmin brain SPECT for the detection of recurrent high-grade glioma. Magn Reson Imaging. 2014;32(7):854-9.

| 1st author | Year | Type of study | Patient number | Type of patients |
| --- | --- | --- | --- | --- |
| Alexiou GA | 2014 | Clinical study | 30 | glioma |
| Intervention | A prospective study was made of 30 patients treated for high-grade glioma who had suspected recurrent tumor on follow-up MRI. All had been treated by surgical resection of the tumor followed by standard postoperative radiotherapy with chemotherapy. No residual tumor had been found on brain imaging immediately after the initial treatment. All the patients were studied with dynamic susceptibility contrast brain MRI and, within a week, 99mTc-Tetrofosmin brain SPECT. | | | |
| Outcome | Both 99mTc-Tetrofosmin brain SPECT and dynamic susceptibility contrast MRI could discriminate between tumor**recurrence**and treatment induced necrosis with 100% sensitivity and 100% specificity. An apparent diffusion coefficient (ADC) ratio cut-off value of 1.27 could differentiate**recurrence**from treatment induced necrosis with 65% sensitivity and 100% specificity and a fractional anisotropy (FA) ratio cut-off value of 0.47 could differentiate**recurrence**from treatment induced necrosis with 57% sensitivity and 100% specificity. A significant correlation was demonstrated between 99mTc-Tetrofosmin uptake ratio and rCBV (P=0.003). | | | |
| Conclusion | Dynamic susceptibility contrast MRI and brain SPECT with 99mTc-Tetrofosmin had the same accuracy and may be used to detect**recurrent**tumor following treatment for**glioma**. DTI also showed promise for the detection of**recurrent**tumor, but was inferior to both dynamic susceptibility contrast MRI and brain SPECT. | | | |

#7.
Sherif M.F., Salem F.M., Almahallawy M.A., Algawad A.M.A., Hammad Q.M.

[Role of magnetic resonance spectroscopy in differentiation between recurrence of glioma and post radiation injury](http://www.embase.com.ezp-prod1.hul.harvard.edu/search/results?subaction=viewrecord&rid=5&page=2&id=L602175378). Egyptian J Radiol Nucl Med. 2014;45(4):1233-1240.

| 1st author | Year | Type of study | Patient number | Type of patients |
| --- | --- | --- | --- | --- |
| Sherif MF | 2014 | Clinical study | 32 | glioma |
| Intervention | 32 patients (20 males and 12 females) complaining of different neurological symptoms were enrolled prospectively in this study between September 2011 and December 2013. These patients were selected on the basis that they were known patients with pathologically proved glioma who underwent radiotherapy. All patients underwent standard MRI examination and MR spectroscopy. | | | |
| Outcome | This study included 32 patients, twenty four patients (75%) proved histologically to be of**recurrent glioma**(group I) and 8 cases (25%) diagnosed as post radiation injury (group II). Peri-tumoral infiltration was present in 18 cases (56.25%) of**recurrent glioma**. Significantly increased Cho/Cr and Cho/NAA ratios were observed in neoplastic (n = 24) compared with non-neoplastic lesions (n = 8). Presence of lactate and lipid yielded correct classification as neoplastic and non-neoplastic lesions. | | | |
| Conclusion | Magnetic resonance spectroscopy is a useful tool for the detection of**recurrent glioma**and differentiation from post radiation injury. | | | |

#8.
D'Souza MM, Sharma R, Jaimini A, Panwar P, Saw S, Kaur P, et al. 11C-MET PET/CT and advanced MRI in the evaluation of tumor recurrence in high-grade gliomas. Clin Nucl Med. 2014;39(9): 791-798.

| 1st author | Year | Type of study | Patient number | Type of patients |
| --- | --- | --- | --- | --- |
| D’Souza MM | 2014 | Clinical study | 29 | glioma |
| Intervention | Twenty-nine patients with high-grade gliomas who underwent surgical resection, external beam radiation therapy, and standard regimens of chemotherapy were subjected to MRI (conventional, MR perfusion, and MR spectroscopy) and C-MET PET/CT scans. A definitive diagnosis was made based on histopathology and/or long-term clinical and radiological follow-up. Several indices were obtained for lesion characterization, namely, SUVmean, SUVmax, and mean lesion-to-normal tissue on PET/CT, as well as relative cerebral blood volume and choline-to-creatine ratio on MRI. | | | |
| Outcome | Histological examination revealed viable tumor cells in 19 cases, whereas the remaining 10 were deemed to be negative based on histology (3 cases) or long-term follow-up (7 cases). All the quantitative indices mentioned previously tended to be higher in patients with tumor**recurrence**/residual. The sensitivity, specificity, and accuracy of C-MET PET/CT in identifying tumor**recurrence**/residual were 94.7%, 80%, and 89.6%, respectively, whereas that of MRI were 84.2%, 90%, and 86.2%, respectively. | | | |
| Conclusion | Both C-MET PET/CT and MRI (with the inclusion of advanced MRI techniques) demonstrated a high diagnostic performance in the identification of tumor residual/**recurrence**in high-grade gliomas posttherapy. Although C-MET PET/CT seemed to be more sensitive, whereas advanced MRI seemed more specific, there was no statistically significant difference in the diagnostic performance of either modality in the present study. Further studies with a larger group of patients are warranted. | | | |

**16 excluded references and reasons for being excluded (marked in red)**

1. Chung WJ, Kim HS, Kim N, Choi CG, Kim SJ. Recurrent glioblastoma: optimum area under the curve method derived from dynamic contrast-enhanced T1-weighted perfusion MR imaging. Radiology. 2013;269(2): 561-568.

[Provide no outcome of interest]

1. Reddy K, Westerly D, Chen C. MRI patterns of T1 enhancing radiation necrosis versus tumour recurrence in high-grade gliomas. J Med Imaging Radiat Oncol. 2013;57(3): 349-355.

[Being one-arm study]

1. Elias AE, Carlos RC, Smith EA, Frechtling D, George B, Maly P, et al. MR spectroscopy using normalized and non-normalized metabolite ratios for differentiating recurrent brain tumor from radiation injury. Acad Radiol. 2011;18(9): 1101-1108.

[Being one-arm study]

1. Bobek-Billewicz B, Stasik-Pres G, Majchrzak H, Zarudzki L. Differentiation between brain tumor recurrence and radiation injury using perfusion, diffusion-weighted imaging and MR spectroscopy. Folia Neuropathol. 2010;48(2): 81-92.

[Provide no outcome of interest]

1. Rabinov JD, Lee PL, Barker FG, Louis DN, Harsh GR, Cosgrove GR, et al. In vivo 3-T MR spectroscopy in the distinction of recurrent glioma versus radiation effects: initial experience. Radiology. 2002;225(3): 871-879.

[Being one-arm study]

1. Lai PH, Ho JT, Chen WL, Hsu SS, Wang JS, Pan HB, et al. Brain abscess and necrotic brain tumor: discrimination with proton MR spectroscopy and diffusion-weighted imaging. AJNR Am J Neuroradiol. 2002;23(8): 1369-1377.

[Being one-arm study]

1. Taylor JS, Langston JW, Reddick WE, Kingsley PB, Ogg RJ, Pui MH, et al. Clinical value of proton magnetic resonance spectroscopy for differentiating recurrent or residual brain tumor from delayed cerebral necrosis. Int J Radiat Oncol Biol Phys. 1996;36(5): 1251-1261.

[Provide no outcome of interest]

1. Ikehira H, Miyamoto T, Yasukawa T, Obata T, Katoh H, Koga M, et al. Differences in metabolic and morphological reactions after radiation therapy: proton NMR spectroscopy and imaging of patients with intracranial tumors. Radiat Med. 1995;13(5): 199-204.

[Being one-arm study]

1. Shah R, Vattoth S, Jacob R, Manzil FF, O'Malley JP, Borghei P, et al. Radiation necrosis in the brain: imaging features and differentiation from tumor recurrence. Radiographics. 2012;32(5): 1343-1359.

[Not a clinical study, so provide no outcome of interest]

1. Narang J, Jain R, Arbab AS, Mikkelsen T, Scarpace L, Rosenblum ML, et al. Differentiating treatment-induced necrosis from recurrent/progressive brain tumor using nonmodel-based semiquantitative indices derived from dynamic contrast-enhanced T1-weighted MR perfusion. Neuro Oncol 2011;13(9): 1037-1046.

[Provide no outcome of interest]

1. Amin A, Moustafa H, Ahmed E, El-Toukhy M. Glioma residual or recurrence versus radiation necrosis: accuracy of pentavalent technetium-99m-dimercaptosuccinic acid [Tc-99m (V) DMSA] brain SPECT compared to proton magnetic resonance spectroscopy (1H-MRS): initial results. J Neurooncol. 2012;106(3): 579-587.

[Provide no outcome of interest]

1. Nakajima T, Kumabe T, Kanamori M, Saito R, Tashiro M, Watanabe M, et al. Differential diagnosis between radiation necrosis and glioma progression using sequential proton magnetic resonance spectroscopy and methionine positron emission tomography. Neurol Med Chir (Tokyo). 2009;49(9): 394-401.

[Unclear grouping description or being one-arm study]

1. Boxerman JL, Ellingson BM, Jeyapalan S, Elinzano H, Harris RJ, Rogg JM, et al. Longitudinal DSC-MRI for distinguishing tumor recurrence from pseudoprogression in patients with a high-grade glioma. Am J Clin Oncol. 2014 Nov 26. [Epub ahead of print].

[Provide no outcome of interest]

1. Kim DY, Kim HS, Goh MJ, Choi CG, Kim SJ. Utility of intravoxel incoherent motion MR imaging for distinguishing recurrent metastatic tumor from treatment effect following gamma knife radiosurgery: initial experience. AJNR Am J Neuroradiol. 2014;35(11): 2082-2090.

[Provide no outcome of interest]

1. Sherif M.F., Salem F.M., Almahallawy M.A., Algawad A.M.A., Hammad Q.M.

[Role of magnetic resonance spectroscopy in differentiation between recurrence of glioma and post radiation injury](http://www.embase.com.ezp-prod1.hul.harvard.edu/search/results?subaction=viewrecord&rid=5&page=2&id=L602175378). Egyptian J Radiol Nucl Med. 2014;45(4):1233-1240.
[Not a clinical study, so it provided no outcome of interest]

1. Ratai EM, Zhang Z, Snyder BS, Boxerman JL, Safriel Y, McKinstry RC,et al. Magnetic resonance spectroscopy as an early indicator of response to anti-angiogenic therapy in patients with recurrent glioblastoma: RTOG 0625/ACRIN 6677. Neuro Oncol. 2013;15(7):936-44.

[Provide no outcome of interest]

**Study Protocol & Instruction for Abstract Search and Full-text Review**

**Tentative topic:** Differentiating treatment-induced necrosis from recurrent brain tumor using MR perfusion and MR spectroscopy: a meta-aqnalysis.

**Objective:** The aim of our study is to evaluate the diagnostic effectiveness of MR perfusion and MR spectroscopy in the differentiation of the tumor recurrence from treatment-induced necrosis or radiation related injury. The study can be designed for: (1) comparison between treatment-induced necrosis and recurrent brain tumor in rCBV, rPH, PSR, L/N ratio and/or ADC; (2) comparison between MR perfusion and MR spectroscopy in sensitivity and specificity for identifying recurrent metastatic tumors.

**Background:**

Differentiating treatment-induced necrosis (TIN; e.g. radiation necrosis) from recurrent/progressive tumor (RPT) is a common yet challenging task in a busy neuro-oncologic practice. The importance of differentiating these 2 entities cannot be overemphasized, as the treatment options and prognosis for the 2 are different. With the advent of newer aggressive treatment options for brain tumors, including various combination neoadjuvant therapeutic strategies, the follow-up imaging appearance of a previously treated brain tumor is also becoming more and more complex. There have been various attempts to differentiate these 2 entities in the past using conventional morphologic imaging as well as various functional imaging techniques such as CT perfusion, MR perfusion, diffusion weighted imaging (DWI), MR spectroscopy, and single-photon emission computed tomography and PET. However, each modality has its limitations, and the search for an easy to use yet accurate modality continues [Narang et al., 2011]. Conventional magnetic resonance (MR) imaging does not provide sufficient information to differentiate delayed radiation effects from tumor recurrence. Positron emission tomography, MR spectroscopy, and other modalities sometimes may lead to false findings of tumor recurrence [Mitsuya et al., 2010].

The relative cerebral blood volume (rCBV), relative peak height (rPH), percentage of signal-intensity recovery (PSR), Lesion to normal (L/N) or apparent diffusion coefficient (ADC) derived from MR perfusion and MR spectroscopy imaging should be calculated and used for distinguishing recurrent metastatic tumor from radiation necrosis. The sensitivity and specificity of the modality for identifying recurrent metastatic tumors should be compared as well.

We hypothesized that imaging from MR perfusion and MR spectroscopy has the potential to differentiate recurrent or progressive tumor growth from treatment-induced damage/necrosis to brain tissue after radiation therapy.

The inclusion criteria may include: (a) They received a new diagnosis of GBM on the basis of MR findings in the brain; *(b)* they underwent imageguided gross total tumor resection of the enhancing component; *(c)* they underwent treatment with postsurgical conventional EBRT; *(d)* they subsequently developed a progressively enlarging region of contrast enhancement within the radiation field, as indicated by two or more consecutive MR examinations with findings that were suggestive of GBM recurrence or radiation necrosis; *(e)* they underwent perfusion MR imaging of the progressively enhancing region [Barajas et al., 2009].

**Keywords**: magnetic resonance spectroscopy/MR spectroscopy, magnetic resonance perfusion/MR perfusion, brain tumors, recurrence, radiation injury/ radiation necrosis

**Review procedure**

*Inclusion criteria*:

- RCTs or two-arm prospective studies; Retrospective studies can be included;
- Patients with brain tumors and previously operated upon and treated with radiotherapy; The lessions were examined with perfusion MR imaging or MR spectroscopy;
- Quantitative outcomes include any of the following parameters: The relative cerebral blood volume (rCBV), relative peak height (rPH), percentage of signal-intensity recovery (PSR), Lesion to normal (L/N) or apparent diffusion coefficient (ADC)

*Exclusion criteria*:

- Cohort study; Letters, comments, editorials, Case report; proceeding, personal communication;
- Patients with brain tumors but treated with surgery ressection only, or chemotherapy only;
- The lesions were examined with CT, PET and/or DWI only;
- No quantitative primary outcome.

Major database for search:

- MEDLINE: http://www.ncbi.nlm.nih.gov/pubmed
- CENTRAL: The Cochrane Central Register of Controlled Trials ([http://www.thecochranelibrary.com](http://www.thecochranelibrary.com/).)
- EMBASE & Google Scholars
- Hand searching: reference in relevant articles

**Tables for search outcome:**

1. Table 1. Searching process
2. Table 2. Study characteristics

Table 1. Searching process

| Database | Key words and the combination | Articles found (N) | Articles excluded (N) | Qualified articles (N) |
| --- | --- | --- | --- | --- |
| MEDLINE |  |  |  |  |
| CENTRAL |  |  |  |  |
| EMBASE |  |  |  |  |
| Other databases |  |  |  |  |
| Hand searching |  |  |  |  |
| Total | |  |  |  |

Table 2. Study characteristics

| 1st author | Year | Type of study | Patient number | Type of patients |
| --- | --- | --- | --- | --- |
|  |  |  |  |  |
| Intervention |  | | | |
| Outcome |  | | | |
| Conclusion |  | | | |

**References**:

***Meta-analysis& systematic review***

Hollingworth W, [Medina LS](http://www.ncbi.nlm.nih.gov/pubmed?term=Medina LS%5BAuthor%5D&cauthor=true&cauthor_uid=16908548), [Lenkinski RE](http://www.ncbi.nlm.nih.gov/pubmed?term=Lenkinski RE%5BAuthor%5D&cauthor=true&cauthor_uid=16908548), [Shibata DK](http://www.ncbi.nlm.nih.gov/pubmed?term=Shibata DK%5BAuthor%5D&cauthor=true&cauthor_uid=16908548), [Bernal B](http://www.ncbi.nlm.nih.gov/pubmed?term=Bernal B%5BAuthor%5D&cauthor=true&cauthor_uid=16908548), [Zurakowski D](http://www.ncbi.nlm.nih.gov/pubmed?term=Zurakowski D%5BAuthor%5D&cauthor=true&cauthor_uid=16908548), et al. A systematic literature review of magnetic resonance spectroscopy for the characterization of brain tumors. [AJNR Am J Neuroradiol.](http://www.ncbi.nlm.nih.gov/pubmed/16908548) 2006;27(7):1404-11.

***Clinical reports***

*MR perfusion*

Shah R, Vattoth S, Jacob R, Manzil FF, O'Malley JP, Borghei P, et al. Radiation necrosis in the brain: imaging features and differentiation from tumor recurrence. Radiographics. 2012;32(5): 1343-1359.

Narang J, Jain R, Arbab AS, Mikkelsen T, Scarpace L, Rosenblum ML, et al. Differentiating treatment-induced necrosis from recurrent/progressive brain tumor using nonmodel-based semiquantitative indices derived from dynamic contrast-enhanced T1-weighted MR perfusion. Neuro Oncol 2011;13(9): 1037-1046.

Bobek-Billewicz B, Stasik-Pres G, Majchrzak H, Zarudzki L. Differentiation between brain tumor recurrence and radiation injury using perfusion, diffusion-weighted imaging and MR spectroscopy. Folia Neuropathol. 2010;48(2): 81-92.

Mitsuya K, Nakasu Y, Horiguchi S, Harada H, Nishimura T, Bando E, et al. Perfusion weighted magnetic resonance imaging to distinguish the recurrence of metastatic brain tumors from radiation necrosis after stereotactic radiosurgery. J Neurooncol. 2010;99(1): 81-88.

Barajas RF, Chang JS, Sneed PK, Segal MR, McDermott MW, Cha S, et al. Distinguishing recurrent intra-axial metastatic tumor from radiation necrosis following gamma knife radiosurgery using dynamic susceptibility-weighted contrast-enhanced perfusion MR imaging. AJNR Am J Neuroradiol. 2009;30(2): 367-372.

*MR spectroscopy*

Amin A, Moustafa H, Ahmed E, El-Toukhy M. Glioma residual or recurrence versus radiation necrosis: accuracy of pentavalent technetium-99m-dimercaptosuccinic acid [Tc-99m (V) DMSA] brain SPECT compared to proton magnetic resonance spectroscopy (1H-MRS): initial results. J Neurooncol. 2012;106(3): 579-587.

Nakajima T, Kumabe T, Kanamori M, Saito R, Tashiro M, Watanabe M, et al. Differential diagnosis between radiation necrosis and glioma progression using sequential proton magnetic resonance spectroscopy and methionine positron emission tomography. Neurol Med Chir (Tokyo). 2009;49(9): 394-401.

Schlemmer HP, Bachert P, Henze M, Buslei R, Herfarth KK, Debus J, et al. [Differentiation of radiation necrosis from tumor progression using proton magnetic resonance spectroscopy.](http://www.ncbi.nlm.nih.gov/pubmed/11942375) Neuroradiology. 2002;44(3):216-22.

Mitsuya K, Nakasu Y, Horiguchi S, Harada H, Nishimura T, Bando E, et al. Perfusion weighted magnetic resonance imaging to distinguish the recurrence of metastatic brain tumors from radiation necrosis after stereotactic radiosurgery. J Neurooncol. 2010;99(1): 81-88.
