# Supplementary material for: Differentiating Radiation-Induced Necrosis from Recurrent Brain Tumor Using MR Perfusion and Spectroscopy: A Meta-Analysis
Source: PLoS One. 2016 Jan 7;11(1):e0141438. doi: 10.1371/journal.pone.0141438 (PMC4712150; doi:10.1371/journal.pone.0141438)
Supplement: S1 PRISMA Checklist — (DOC) [file pone.0141438.s002.doc]

**PRISMA CHECKLIST**

| **Section/topic** | **#** | **Checklist item** | **Reported on page #** |
| --- | --- | --- | --- |
| **TITLE** | | |  |
| Title | 1 | Identify the report as a systematic review, meta-analysis, or both.  **Differentiating radiation-induced necrosis from recurrent brain tumor using MR perfusion and spectroscopy: a meta-analysis** | 1 |
| **ABSTRACT** | | |  |
| Structured summary | 2 | Provide a structured summary including, as applicable: background; objectives; data sources; study eligibility criteria, participants, and interventions; study appraisal and synthesis methods; results; limitations; conclusions and implications of key findings; systematic review registration number.  **Purpose:** This meta-analysis examined roles of several metabolites in differentiating recurrent tumor from necrosis in patients with brain tumors using MR perfusion and spectroscopy.  **Methods:** Medline, Cochrane, EMBASE, and Google Scholar were searched for studies using perfusion MRI and/or MR spectroscopy published up to March 4, 2015 which differentiated between recurrent tumor vs. necrosis in patients with primary brain tumors or brain metastasis. Only two-armed, prospective or retrospective studies were included. A meta-analysis was performed on the difference in relative cerebral blood volume (rCBV), ratios of choline/creatine (Cho/Cr) and/or choline/N-acetyl aspartate (Cho/NAA) between participants undergoing MRI evaluation. A χ2 based test of homogeneity was performed using Cochran’s Q statistic and I2.  **Results:** Of 397 patients in 13 studies who were analyzed, the majority had tumor recurrence. As there was evidence of heterogeneity among 10 of the studies which used rCBV for evaluation (Q statistic = 31.634, I2 = 97.11%, P < 0.0001) a random-effects analysis was applied. The pooled difference in means (2.18, 95%CI = 0.85 to 3.50) indicated that the average rCBV in a contrast-enhancing lesion was significantly higher in tumor recurrence compared with radiation injury (P = 0.001). Based on a fixed-effect model of analysis encompassing the six studies which used Cho/Cr ratios for evaluation (Q statistic = 8.388, I2 = 40.39%, P = 0.137), the pooled difference in means (0.77, 95%CI = 0.57 to 0.98) of the average Cho/Cr ratio was significantly higher in tumor recurrence than in tumor necrosis (P = 0.001). There was significant difference in ratios of Cho to NAA between recurrent tumor and necrosis (1.02, 95%CI = 0.03 to 2.00, P = 0.044).  **Conclusions:** MR spectroscopy and MR perfusion using Cho/NAA and Cho/Cr ratios and rCBV may increase the accuracy of differentiating necrosis from recurrent tumor in patients with primary brain tumors or metastases. | 2 |
| **INTRODUCTION** | | |  |
| Rationale | 3 | Describe the rationale for the review in the context of what is already known.  Differentiating radiation necrosis from recurrent/progressive tumor is an important but challenging task, as the treatment options and prognosis for each is different. To differentiate between the two, surgical biopsy with re-operation is often required to be certain of the diagnosis before further management can be planned.  It is for this reason that recent studies have investigated the use of more advanced imaging methods that are able to monitor physiological and metabolic properties of tumor [2]. These functional imaging techniques include CT perfusion, MR perfusion [11-15], diffusion weighted imaging (DWI)[2, 3], MR spectroscopy [12, 14, 16-22] single-photon emission computed tomography (SPECT) [23, 24], and positron emission tomography (PET) [25-27]. However, each modality has its limitations. Conventional MRI does not provide sufficient information to differentiate delayed radiation effects from tumor recurrence, whereas PET, MR spectroscopy, and other modalities can lead to false positive findings of tumor recurrence [13].  Although the gold standard is still brain biopsy, high levels of choline (Cho) are often observed in areas with high cellular membrane turnover, and relatively increased cerebral blood volume (rCBV) reflects tumor neovascularization [11-15]. N-acetyl aspartate (NAA) is another metabolite found in neurons, and creatine (Cr) is rich in regions active with energy metabolism. A scoring system using these parameters may increase the accuracy of differentiating recurrent tumor tissues from necrosis caused by delayed radiation effects. | 4,5 |
| Objectives | 4 | Provide an explicit statement of questions being addressed with reference to participants, interventions, comparisons, outcomes, and study design (PICOS).  The aim of our study was to evaluate the diagnostic effectiveness of MR perfusion and MR spectroscopy in differentiating recurrent tumor from necrosis caused by radiation, based on parameters such as rCBV and ratios of choline/creatine (Cho/Cr ) and choline/N-acetyl aspartate (Cho/NAA). We hypothesized that imaging from MR perfusion and MR spectroscopy has the potential to differentiate recurrent or progressive tumor growth from treatment-induced necrosis in brain tissue after radiation therapy. | 5 |
| **METHODS** | | |  |
| Protocol and registration | 5 | Indicate if a review protocol exists, if and where it can be accessed (e.g., Web address), and, if available, provide registration information including registration number. | N/A |
| Eligibility criteria | 6 | Specify study characteristics (e.g., PICOS, length of follow-up) and report characteristics (e.g., years considered, language, publication status) used as criteria for eligibility, giving rationale.  We included only two-armed (recurrent tumor vs. necrosis) prospective or retrospective studies of patients with primary brain tumors or brain metastasis evaluated using MR perfusion or MR spectroscopy, or both. The study design had to involve at least one of the outcome measures, i.e., relative cerebral blood volume (rCBV), ratio of Cho/Cr and/or ratio of Cho/NAA. Only English language publications were included.  Letters, comments, editorials, case reports, proceedings, and personal communications were excluded. In addition, any study design which did not contain at least one of the quantitative primary or secondary outcome measures was also excluded.  ***Search strategy***  Searched databases included Medline, Cochrane, EMBASE, and Google Scholar which were searched until March 4, 2015. The reference lists of relevant studies were hand-searched. Keywords used for the search included magnetic resonance spectroscopy/MR spectroscopy, magnetic resonance perfusion/MR perfusion, brain tumors, brain metastasis, recurrence, radiation injury/radiation necrosis. | 6 |
| Information sources | 7 | Describe all information sources (e.g., databases with dates of coverage, contact with study authors to identify additional studies) in the search and date last searched.  Searched databases included Medline, Cochrane, EMBASE, and Google Scholar which were searched until March 4, 2015. | 6 |
| Search | 8 | Present full electronic search strategy for at least one database, including any limits used, such that it could be repeated.  The reference lists of relevant studies were hand-searched. Keywords used for the search included magnetic resonance spectroscopy/MR spectroscopy, magnetic resonance perfusion/MR perfusion, brain tumors, brain metastasis, recurrence, radiation injury/radiation necrosis.  Studies were identified by two independent reviewers using the search strategy. Where there was uncertainty regarding eligibility, a third reviewer was consulted.  The following data were extracted from studies that met the inclusion criteria: the name of the first author, year of publication, study design, number of participants in each treatment group, participants’ age and gender, patients’ type, primary and secondary outcomes, as well as time of follow-up. | 6 |
| Study selection | 9 | State the process for selecting studies (i.e., screening, eligibility, included in systematic review, and, if applicable, included in the meta-analysis).  We included only two-armed (recurrent tumor vs. necrosis) prospective or retrospective studies of patients with primary brain tumors or brain metastasis evaluated using MR perfusion or MR spectroscopy, or both. The study design had to involve at least one of the outcome measures, i.e., relative cerebral blood volume (rCBV), ratio of Cho/Cr and/or ratio of Cho/NAA. Only English language publications were included.  Studies were identified by two independent reviewers using the search strategy. Where there was uncertainty regarding eligibility, a third reviewer was consulted.  The following data were extracted from studies that met the inclusion criteria: the name of the first author, year of publication, study design, number of participants in each treatment group, participants’ age and gender, patients’ type, primary and secondary outcomes, as well as time of follow-up. | 6 |
| Data collection process | 10 | Describe method of data extraction from reports (e.g., piloted forms, independently, in duplicate) and any processes for obtaining and confirming data from investigators.  The reference lists of relevant studies were hand-searched. Studies were identified by two independent reviewers using the search strategy. Where there was uncertainty regarding eligibility, a third reviewer was consulted. | 6 |
| Data items | 11 | List and define all variables for which data were sought (e.g., PICOS, funding sources) and any assumptions and simplifications made.  The following data were extracted from studies that met the inclusion criteria: the name of the first author, year of publication, study design, number of participants in each treatment group, participants’ age and gender, patients’ type, primary and secondary outcomes, as well as time of follow-up. | 6 |
| Risk of bias in individual studies | 12 | Describe methods used for assessing risk of bias of individual studies (including specification of whether this was done at the study or outcome level), and how this information is to be used in any data synthesis.  Publication bias analysis was not performed because the number of studies was too few to detect an asymmetric funnel [30]. | 7 |
| Summary measures | 13 | State the principal summary measures (e.g., risk ratio, difference in means).  The outcomes for this meta-analysis were the difference in rCBV and ratios of Cho/Cr and Cho/NAA between tumor recurrence and radiation necrosis. If the median and interquartile range (IQR) is reported in a study, assumption that the median of the outcome variable is equal to the mean response would be made and width of the interquartile range would be approximately 1.35 times standard deviation [28]. If the data lacked in mean and standard deviation, median, range, and size of the sample were used to estimate the mean and variance [29]. The difference in means with 95% CI was calculated for each individual study and for those studies pooled. A χ2-based test of homogeneity was performed and the inconsistency index (I^2^) and Q statistics were determined. If the I^2^ statistic was > 50%, a random-effects model was used. Otherwise, fixed-effects models were employed. Pooled effects were calculated and a two-sided P value < 0.05 was considered statistically significant.  Sensitivity analysis was carried out for the outcomes using the leave one-out approach. Publication bias analysis was not performed because the number of studies was too few to detect an asymmetric funnel [30]. All analyses were performed using Comprehensive Meta-Analysis Statistical Software, version 2.0 (Biostat, Englewood, NJ, USA). | 7 |
| Synthesis of results | 14 | Describe the methods of handling data and combining results of studies, if done, including measures of consistency (e.g., I^2^) for each meta-analysis.  The difference in means with 95% CI was calculated for each individual study and for those studies pooled. A χ2-based test of homogeneity was performed and the inconsistency index (I^2^) and Q statistics were determined. If the I^2^ statistic was > 50%, a random-effects model was used. Otherwise, fixed-effects models were employed. Pooled effects were calculated and a two-sided P value < 0.05 was considered statistically significant. | 7 |

| **Section/topic** | **#** | **Checklist item** | **Reported on page #** |
| --- | --- | --- | --- |
| Risk of bias across studies | 15 | Specify any assessment of risk of bias that may affect the cumulative evidence (e.g., publication bias, selective reporting within studies).  ***Publication Bias***  Publication bias regarding outcomes was not assessed because there were fewer than ten studies required to detect funnel plot asymmetry [30]. | 10 |
| Additional analyses | 16 | Describe methods of additional analyses (e.g., sensitivity or subgroup analyses, meta-regression), if done, indicating which were pre-specified. A χ2-based test of homogeneity was performed and the inconsistency index (I^2^) and Q statistics were determined. If the I^2^ statistic was > 50%, a random-effects model was used. Otherwise, fixed-effects models were employed. Pooled effects were calculated and a two-sided P value < 0.05 was considered statistically significant.  Sensitivity analysis was carried out for the outcomes using the leave one-out approach. Publication bias analysis was not performed because the number of studies was too few to detect an asymmetric funnel [30]. All analyses were performed using Comprehensive Meta-Analysis Statistical Software, version 2.0 (Biostat, Englewood, NJ, USA). | 7 |
| **RESULTS** | | |  |
| Study selection | 17 | Give numbers of studies screened, assessed for eligibility, and included in the review, with reasons for exclusions at each stage, ideally with a flow diagram.  A total of 37 articles were reviewed based on eligibility, and among them, nine were excluded based on the inclusion/exclusion criteria. After full text review of the remaining 28 studies, 15 studies were excluded due to lack of outcomes of interest (n = 10) or only one-arm study design (n = 5), as shown in our flow chart for study selection (Fig. 1).  The remaining 13 articles [11-18,31-35] evaluating patients with primary brain tumors or brain metastasis for recurrent tumor vs. necrosis using MR perfusion or MR spectroscopy were used for the meta-analysis. The characteristics of all 13 studies are summarized in Table 1. | 8 |
| Study characteristics | 18 | For each study, present characteristics for which data were extracted (e.g., study size, PICOS, follow-up period) and provide the citations.  As shown in Table 1, six studies [11,13,18,31-33] were prospective and seven [12,14-17,34,35] were retrospective two-armed studies. The 397 patients encompassed by the 13 studies (Table 1) had an average age ranging from 34 years to 63 years and the majority of patients were male. The majority of patients also had recurrent tumor rather than radiation necrosis. Of the seven studies [11,14,16,18,32-34] which provided WHO grades, most tumors were grades II-IV.  The outcomes of all included studies are summarized in Table 2. Ten studies [11-15,31-35] evaluated the efficacy of using MR perfusion to differentiate recurrent tumor vs. necrosis, six studies used MR spectroscopy [12,14,16, 18,32,33], and only four studies [12, 14,32,33] used both MR spectroscopy and MR perfusion to differentiate radiation necrosis from tumor recurrence.  ***Difference of rCBV in tumor vs. necrosis***  Fig. 2A is a forest plot of the difference in means of rCBV. Ten [11-15, 31-35] of the 13 studies provided complete numerical data evaluating rCBV for recurrent tumor vs. necrosis, and were included in the meta-analysis. There was evidence of heterogeneity regarding the rCBV values among the 10 studies (Q statistic = 311.634, I^2^ = 97.11%, P < 0.001); therefore, a random-effects model of analysis was used. The pooled difference in means (2.18, 95%CI = 0.85 to 3.50) indicated the mean rCBV in a contrast-enhancing lesion was significantly higher in tumor recurrence compared with radiation injury (P = 0.001, Fig. 2A).  ***Ratios of Cho/Cr and Cho/NAA in tumor vs. necrosis***  Fig. 2B is a forest plot of the difference in means of the Cho/Cr ratio. Six [12, 14, 16, 18,32,33] of the 13 studies showed no evidence of heterogeneity regarding Cho/Cr ratio evaluation and were included in the meta-analysis using a fixed-effect model of analysis (Q statistic = 8.388, I^2^ = 40.39%, P = 0.137). The pooled difference in means (0.77, 95%CI = 0.57 to 0.98) indicated the mean Cho/Cr ratio was significantly higher in tumor recurrence than in necrosis (P = 0.000, Fig. 2B).  Fig. 2C is a forest plot of the difference in means of the Cho/NAA ratio. Four [12, 14, 16,32] of the 13 studies provided completed numerical data regarding Cho/NAA ratio for recurrent tumor vs. necrosis, and were included in the meta-analysis. There was evidence of heterogeneity regarding the Cho/NAA ratio among the four studies (Q statistic = 13.99, I^2^ = 78.55%, P = 0.003); therefore, a random-effects model of analysis was used. There was significant difference in Cho/NAA ratio between recurrent tumor and necrosis (1.02, 95%CI = 0.03 to 2.00, P = 0.044, Fig. 2C). | 8,9 |
| Risk of bias within studies | 19 | Present data on risk of bias of each study and, if available, any outcome level assessment (see item 12). | N/A |
| Results of individual studies | 20 | For all outcomes considered (benefits or harms), present, for each study: (a) simple summary data for each intervention group (b) effect estimates and confidence intervals, ideally with a forest plot.  ***Difference of rCBV in tumor vs. necrosis***  Fig. 2A is a forest plot of the difference in means of rCBV. Ten [11-15, 31-35] of the 13 studies provided complete numerical data evaluating rCBV for recurrent tumor vs. necrosis, and were included in the meta-analysis. There was evidence of heterogeneity regarding the rCBV values among the 10 studies (Q statistic = 311.634, I^2^ = 97.11%, P < 0.001); therefore, a random-effects model of analysis was used. The pooled difference in means (2.18, 95%CI = 0.85 to 3.50) indicated the mean rCBV in a contrast-enhancing lesion was significantly higher in tumor recurrence compared with radiation injury (P = 0.001, Fig. 2A).  ***Ratios of Cho/Cr and Cho/NAA in tumor vs. necrosis***  Fig. 2B is a forest plot of the difference in means of the Cho/Cr ratio. Six [12, 14, 16, 18,32,33] of the 13 studies showed no evidence of heterogeneity regarding Cho/Cr ratio evaluation and were included in the meta-analysis using a fixed-effect model of analysis (Q statistic = 8.388, I^2^ = 40.39%, P = 0.137). The pooled difference in means (0.77, 95%CI = 0.57 to 0.98) indicated the mean Cho/Cr ratio was significantly higher in tumor recurrence than in necrosis (P = 0.000, Fig. 2B).  Fig. 2C is a forest plot of the difference in means of the Cho/NAA ratio. Four [12, 14, 16,32] of the 13 studies provided completed numerical data regarding Cho/NAA ratio for recurrent tumor vs. necrosis, and were included in the meta-analysis. There was evidence of heterogeneity regarding the Cho/NAA ratio among the four studies (Q statistic = 13.99, I^2^ = 78.55%, P = 0.003); therefore, a random-effects model of analysis was used. There was significant difference in Cho/NAA ratio between recurrent tumor and necrosis (1.02, 95%CI = 0.03 to 2.00, P = 0.044, Fig. 2C). | 9 |
| Synthesis of results | 21 | Present results of each meta-analysis done, including confidence intervals and measures of consistency.  ***Difference of rCBV in tumor vs. necrosis***  Fig. 2A is a forest plot of the difference in means of rCBV. Ten [11-15, 31-35] of the 13 studies provided complete numerical data evaluating rCBV for recurrent tumor vs. necrosis, and were included in the meta-analysis. There was evidence of heterogeneity regarding the rCBV values among the 10 studies (Q statistic = 311.634, I^2^ = 97.11%, P < 0.001); therefore, a random-effects model of analysis was used. The pooled difference in means (2.18, 95%CI = 0.85 to 3.50) indicated the mean rCBV in a contrast-enhancing lesion was significantly higher in tumor recurrence compared with radiation injury (P = 0.001, Fig. 2A).  ***Ratios of Cho/Cr and Cho/NAA in tumor vs. necrosis***  Fig. 2B is a forest plot of the difference in means of the Cho/Cr ratio. Six [12, 14, 16, 18,32,33] of the 13 studies showed no evidence of heterogeneity regarding Cho/Cr ratio evaluation and were included in the meta-analysis using a fixed-effect model of analysis (Q statistic = 8.388, I^2^ = 40.39%, P = 0.137). The pooled difference in means (0.77, 95%CI = 0.57 to 0.98) indicated the mean Cho/Cr ratio was significantly higher in tumor recurrence than in necrosis (P = 0.000, Fig. 2B).  Fig. 2C is a forest plot of the difference in means of the Cho/NAA ratio. Four [12, 14, 16,32] of the 13 studies provided completed numerical data regarding Cho/NAA ratio for recurrent tumor vs. necrosis, and were included in the meta-analysis. There was evidence of heterogeneity regarding the Cho/NAA ratio among the four studies (Q statistic = 13.99, I^2^ = 78.55%, P = 0.003); therefore, a random-effects model of analysis was used. There was significant difference in Cho/NAA ratio between recurrent tumor and necrosis (1.02, 95%CI = 0.03 to 2.00, P = 0.044, Fig. 2C). | 9 |
| Risk of bias across studies | 22 | Present results of any assessment of risk of bias across studies (see Item 15).  ***Publication Bias***  Publication bias regarding outcomes was not assessed because there were fewer than ten studies required to detect funnel plot asymmetry [30]. | 10 |
| Additional analysis | 23 | Give results of additional analyses, if done (e.g., sensitivity or subgroup analyses, meta-regression [see Item 16]).  ***Sensitivity analysis***  Sensitivity analysis was performed using the leave-one-out approach in which the meta-analysis of the rCBV and ratios of Cho/Cr and Cho/NAA were performed with each study removed in turn (Table 3). For rCBV and ratio of Cho/Cr, the direction and magnitude of combined estimates did not vary markedly with the removal of the studies, indicating the data was not overly influenced by each study. The four pooled differences in means of Cho/NAA ratios remained significant after each study was removed, in turn, except the removal of Di Costanzo (2014)[32] which changed it to non-significant, indicating Di Costanzo (2014) might influenced the pooled estimate.. | 9,10 |
| **DISCUSSION** | | |  |
| Summary of evidence | 24 | Summarize the main findings including the strength of evidence for each main outcome; consider their relevance to key groups (e.g., healthcare providers, users, and policy makers).  The aim of our study was to evaluate the diagnostic effectiveness of MR perfusion and MR spectroscopy in differentiating recurrent tumor from radiation necrosis. Our meta-analysis showed that both average rCBV and average Cho/Cr and Cho/NAA ratios were significantly higher in tumor recurrence compared with radiation injury (all P < 0.05). We performed sensitivity analysis and tested for homogeneity as part of our study. A χ^2^‑based test of homogeneity was performed using Cochran’s Q statistic and I^2^. The studies which used Cho/Cr ratio to distinguish tumor recurrence from necrosis showed good homogeneity. We also tested for reliability based on sensitivity analysis. Sensitivity analysis using leave-one-out approach evaluated the influence of each study on the pooled estimate for both functional scores. The direction and magnitude of the combined estimates did not change markedly with the exclusion of individual studies, indicating that our meta-analysis had good reliability. | 10 |
| Limitations | 25 | Discuss limitations at study and outcome level (e.g., risk of bias), and at review-level (e.g., incomplete retrieval of identified research, reporting bias).  Our study had several limitations including the limited number of studies available for the meta-analysis. In addition, the operators or observers who evaluated rCBV and other MR spectroscopy data were not blinded to other clinical data. The MR spectroscopy parameters used across different studies were not consistent; for example, different studies used different cut-off values of metabolites for comparison. Future studies using multi-voxel spectroscopy may be needed to determine cut-off values of metabolite ratios. Delayed radiation effects can have a long latency period, as already discussed, and this can skew MR spectroscopy results. The sensitivity of perfusion imaging to artifacts is an additional limitation. Finally, there may have been a selection bias with regards to the studies chosen for the meta-analysis. | 12 |
| Conclusions | 26 | Provide a general interpretation of the results in the context of other evidence, and implications for future research.  In conclusion, based on the results of our meta-analysis, rCBV and ratios of Cho/Cr and Cho/NAA were higher in recurrent tumors than in radiation necrosis. MR spectroscopy and MR perfusion using Cho/NAA and Cho/Cr ratios and rCBV may increase the accuracy of differentiating necrosis from recurrent tumor in patients with primary brain tumors or metastases. | 12 |
| **FUNDING** | | |  |
| Funding | 27 | Describe sources of funding for the systematic review and other support (e.g., supply of data); role of funders for the systematic review. | 2 |
